# Peptides Derived from Angiogenin Regulate Cellular Copper Uptake

**DOI:** 10.3390/ijms22179530

**Published:** 2021-09-02

**Authors:** Giovanni Tabbì, Lorena Maria Cucci, Calogero Pinzino, Alessia Munzone, Tiziano Marzo, Silvia Pizzanelli, Cristina Satriano, Antonio Magrì, Diego La Mendola

**Affiliations:** 1 Institute of Crystallography—National Council of Research—CNR, via Paolo Gaifami 18, 95126 Catania, Italy; giovanni.tabbi@cnr.it; 2 Nano Hybrid BioInterfaces Lab (NHBIL), Department of Chemical Sciences, University of Catania, Viale Andrea Doria 6, 95125 Catania, Italy; lorena.cucci@unict.it; 3 Institute for the Chemistry of OrganoMetallic Compounds (ICCOM), National Council of Research—CNR, via G. Moruzzi 1, 56124 Pisa, Italy; calogero.pinzino@pi.iccom.cnr.it; 4 Aix-Marseille Univesité, 52 Avenue Escadrille Normandie Niemen, 13013 Marseille, France; alessia.munzone@etu.univ-amu.fr; 5 Department of Pharmacy, University of Pisa, via Bonanno Pisano 6, 56126 Pisa, Italy; tiziano.marzo@unipi.it

**Keywords:** electron spin resonance, copper, angiogenesis, peptide, ribonucleases, metal complexes, potentiometry, confocal microscopy

## Abstract

The angiogenin protein (ANG) is one of the most potent endogenous angiogenic factors. In this work we characterized by means of potentiometric, spectroscopic and voltammetric techniques, the copper complex species formed with peptide fragments derived from the N-terminal domain of the protein, encompassing the sequence 1-17 and having free amino, Ang1-17, or acetylated N-terminus group, AcAng1-17, so to explore the role of amino group in metal binding and cellular copper uptake. The obtained data show that amino group is the main copper anchoring site for Ang1-17. The affinity constant values, metal coordination geometry and complexes redox-potentials strongly depend, for both peptides, on the number of copper equivalents added. Confocal laser scanning microscope analysis on neuroblastoma cells showed that in the presence of one equivalent of copper ion, the free amino Ang1-17 increases cellular copper uptake while the acetylated AcAng1-17 strongly decreases the intracellular metal level. The activity of peptides was also compared to that of the protein normally present in the plasma (wtANG) as well as to the recombinant form (rANG) most commonly used in literature experiments. The two protein isoforms bind copper ions but with a different coordination environment. Confocal laser scanning microscope data showed that the wtANG induces a strong increase in intracellular copper compared to control while the rANG decreases the copper signal inside cells. These data demonstrate the relevance of copper complexes’ geometry to modulate peptides’ activity and show that wtANG, normally present in the plasma, can affect cellular copper uptake.

## 1. Introduction

Angiogenin (ANG) is the fifth member of the ribonuclease family (RNase) and, despite having weak catalytic activity (~10^5^–10^6^ lower than that of bovine pancreatic RNase), it is essential for angiogenesis stimulation [1,2]. The widespread expression and localization of angiogenin indicates that its physiological and pathological functions are not limited to the promotion of blood vessels growth.

In the last few years, it has been found that ANG acts as a neurotrophic and neuroprotective factor in neurodegenerative diseases such as Alzheimer’s and Parkinson’s diseases [3,4,5,6,7,8]. Furthermore, the *ang* gene is the first loss-of-function gene identified in patients affected by amyotrophic lateral sclerosis (ALS), suggesting a protective role of the protein towards motoneurons [9,10,11,12].

The precise mechanism by which ANG plays these multiple biological roles is not yet clear. Angiogenin must be internalized in the cells and translocated in the cell nucleus, to exploit its RNase catalytic activity, and explicate angiogenic activity [13,14].

ANG has three distinct functional domains: the RNase catalytic sites, consisting of residues His-13, Lys-40 and His-114; the putative cellular binding site encompassing the sequence 60–68 (KNGNPHREN), and the nuclear translocation site involving residues 31–35 (RRRGL) [2,15]. ANG was originally discovered as a tumor angiogenic factor, but it is present in human tissues, including the plasma and cerebrospinal fluid of healthy individuals [16,17,18]. In physiological conditions, ANG present in the plasma has no proliferative impact. It is therefore crucial to find out which factors may activate and modulate protein activity in different pathophysiological conditions.

Copper ions, which activate and amplify the angiogenic responses evoked by major pro-angiogenic stimuli, including the vascular endothelial growth factor (VEGF) and fibroblast growth factor (FGF) [19], could be among these factors. It is noteworthy that copper is the only mobilized metal going from intra- to extracellular space during the process of angiogenesis [20,21]. Moreover, serum copper levels increase in a wide variety of human cancers as well as in different neurodegenerative diseases [22,23,24,25]. Oxidative-induced inflammation is a common trait as additional cause of various neuronal pathologies [26] and copper is a redox active metal that can generate neurotoxic complexes leading to widespread oxidative stress condition in pathologies such as AD and ALS [27,28]. Beta amyloid (Aβ) can bind copper ions in the synaptic cleft, forming complex species with different coordination modes and redox potentials [29,30], leading to the formation of harmful reactive oxygen species (ROS) [31,32,33]. The triggering of oxidative stress can also induce the intracellular accumulation of copper, favoring lipid peroxidation, apoptosis and the formation of SOD1 aggregates [34]. It is worth noting that the amount the copper in some compartments may arise as in the synaptic cleft is high in relation to the neurotransmission of signals, where the basal level around 3 µM can be rapidly enhanced to 100–250 µM upon the excitatory pulse [35,36] or during angiogenesis process, in which the copper is mobilized from inside to outside cells [21,37]. Therefore, it is reasonable to assume an interaction of copper ions with protein and peptides localized in the synaptic cleft or released during angiogenesis process as Angiogenin.

Moreover, it is evident that an altered copper homeostasis is observed in the pathologies in which abnormal levels of ANG are found and it is known that ANG plays a role in the inhibition of oxidative stress in ALS mouse models [38]. However, at present no evidence has been reported on a possible role of ANG in binding copper as a prevention of oxidative stress in neurodegenerative diseases. It is therefore relevant to consider whether ANG can affect intracellular copper levels and its localization, taking into account that in the cytosol copper is prevalently in the monovalent state.

In endothelial cells, copper can induce ANG expression, affect the intracellular protein distribution and the signaling pathways [39]. Additionally, the copper binding to ANG has been found to increase the protein interaction [40], to modulate protein interaction with actin [41], and to induce pro-angiogenic activity both in differentiated neuroblastoma (SH-SY5Y line) [42] and endothelial (HUVEC line) [43] cells.

The native protein (wtANG), which lacks the free amino terminal group, binds copper by involving His-13 and His-114 residues. On the other hand, the recombinant form (rANG), having an extra methionine as first residue, Met(-1), binds copper through its free N-terminal amino group without the involving of C-terminal domain [44].

Biological active peptides can be designed or derived based on primary sequence from a native protein. In a previous work we synthesized two peptides, Ang1-17 and AcAng1-17, which encompass the sequence 1-17 of the Ang protein with the N-terminal amino group, respectively, free or acetylated, and demonstrated them having different coordination modes of copper ions due to the presence or absence of a free amino group. Accordingly, copper complexes of two peptides can differently affect actin microfilament organization in the cell cytoplasm [45].

In this work, we report the Electron Spin Resonance (ESR) and voltammetric characterization of copper complex species formed by Ang1-17 and AcAng1-17 peptides by exploiting both 1:1 and 2:1 metal-to-ligand (M/L) molar ratios, to elucidate on the copper coordination geometry and reduction potential of different species formed.

The potentiometric, UV-visible (UV-vis) and circular dichroism (CD) characterization has been previously reported only at M/L 1:1 [45]. Furthermore, confocal laser scanning microscopy (LSM) measurements were carried out on SH-SY5Y cells stained with copper sensor1 (CS1) [46], a fluorescent probe of Cu^+^, to investigate the potential ability of angiogenin derived peptide fragments to modulate the level of intracellular copper ions.

The activity of the peptides was assessed at different metal-to-ligand molar ratios and compared with those of wtANG and rANG.

## 2. Results

**Figure 1:**
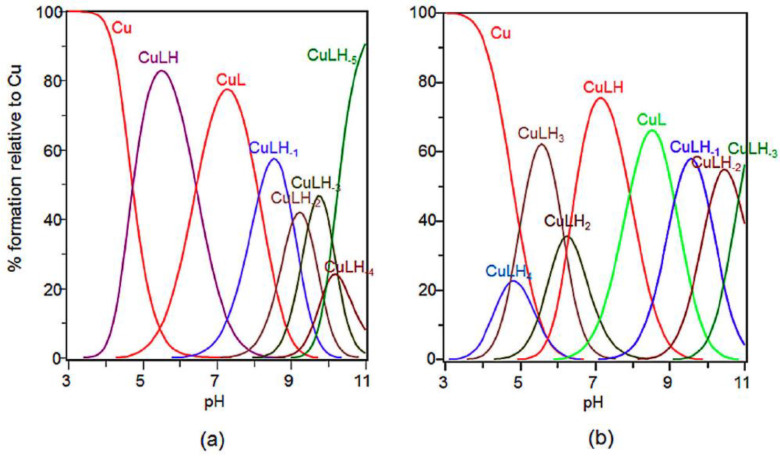
Species distribution of copper(II) complexes with: (**a**) Ang1-17; and (**b**) AcAng1-17. [L] = 1 × 10^−3^ M; metal-to-ligand molar ratio of 1:1. From Ref. [45], under Attribution 4.0 International, CC BY 4.0.

The coordination ability, as well as the distribution of the species as function of the pH values for Ang1-17 and its acetylated form AcAng1-17 with Cu(II) ion at 1:1 metal-to-ligand molar ratio, have been thoroughly investigated in a previous work [45] and the distribution diagrams are reported here for clarity (Figure 1).

### 2.1. ESR Study on Cu(II)-Ang1-17 System (M/L 1:1)

The main species formed by the Cu(II)-Ang1-17 system are [CuLH] and [CuL] in the pH range 5–8.

**Figure 2:**
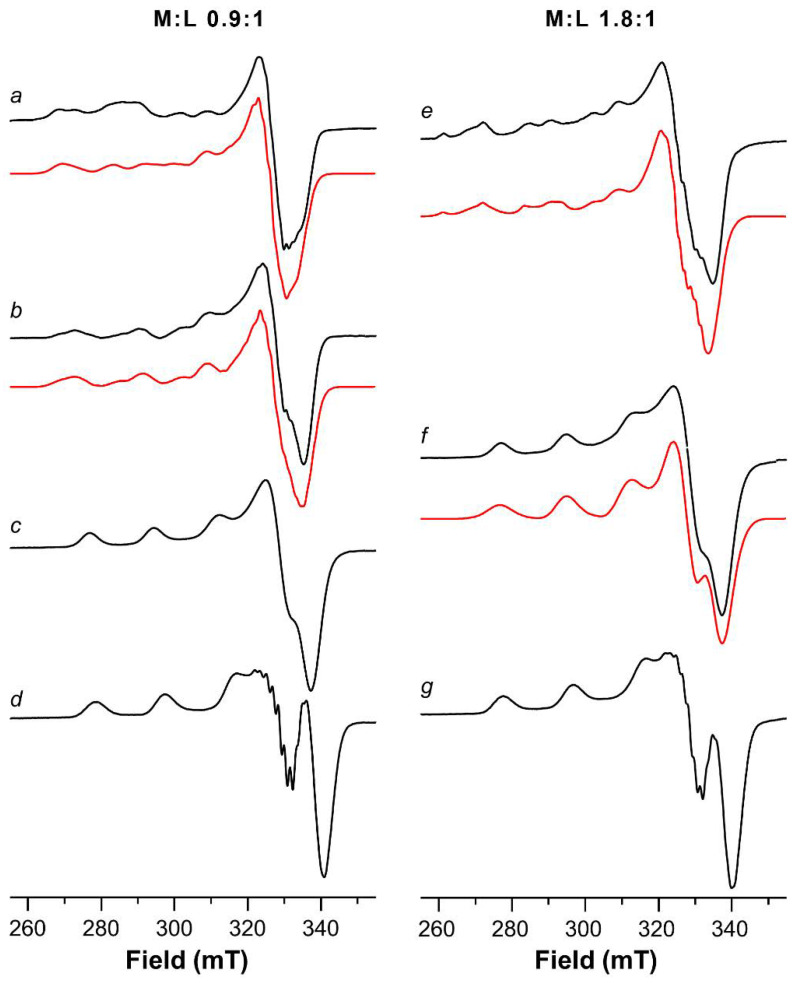
ESR spectra at 150 K of ^63^Cu(II)-Ang1-17 system (**a***–***d**, M:L 0.9:1) and ^63^Cu(II)-Ang1-17 system (e.g., M:L 1.8:1) in aqueous solution as a function of the pH: (**a**) 3.9 subtracted from the [^63^Cu(H_2_O)_6_]^2+^ features; (**b**) 4.5; (**c**) 7.3; (**d**) 10.5; (**e**) 5.5 (subtracted from the spectrum at pH = 6.0); (**f**) 7.4; (**g**) 10.0. Simulated spectra are shown by the red line.

The ESR spectra were run at different pH values to obtain more details about the copper coordination geometry in the different complex species. The Hamiltonian parameters of the [CuLH] isomer species were obtained by simulation of the spectrum run at pH = 3.9 previously subtracted from the Cu(II) hexaaquo ion features (Figure 2a). The Hamiltonian parameters of [CuLH] isomers are g_||_ = 2.335, A_||_ = 146 × 10^−4^ cm^−1^ and g_||_ = 2.295, A_||_ = 176 × 10^−4^ cm^−1^, which contribute roughly equally. In addition, a small amount of a complex having g_||_ = 2.250, A_||_ = 185 × 10^−4^ cm^−1^ was found to also be present at higher a pH.

Raising the pH value to 4.5 (Figure 2b), the [CuLH] isomer (g_||_ = 2.295, A_||_ = 176 × 10^−4^ cm^−1^) continued to persist, but the main species is characterized by g_||_ = 2.250 and A_||_ = 185 × 10^−4^ cm^−1^.

In the 5.3–8.2 pH range, only one species was detected in the ESR spectra (Figure 2c), and the Hamiltonian parameters are characterized by g_||_ = 2.224, A_||_ = 186 × 10^−4^ cm^−1^, drawn directly from the spectrum.

At pH = 9.2 and above, single species spectra (Figure 2d) were recorded, for which the Hamiltonian parameters are invariably g_||_ = 2.194, A_||_ = 201 × 10^−4^ cm^−1^, although a more resolved superfine structure in the perpendicular part of the spectrum is visible at pH = 10.5.

Further deprotonation steps occurring at higher pH values concern the Tyr and Lys residues, which are not directly involved in the Cu(II) coordination.

### 2.2. Potentiometric and ESR Studies on Cu(II)-Ang1-17 System (M/L 2:1)

Potentiometric measurements were carried out by using 2:1 metal-to-ligand molar ratio. As a matter of fact, with the addition of two copper(II) equivalents, only complex species in which the peptide binds two metal ions are observed.

**Figure 3:**
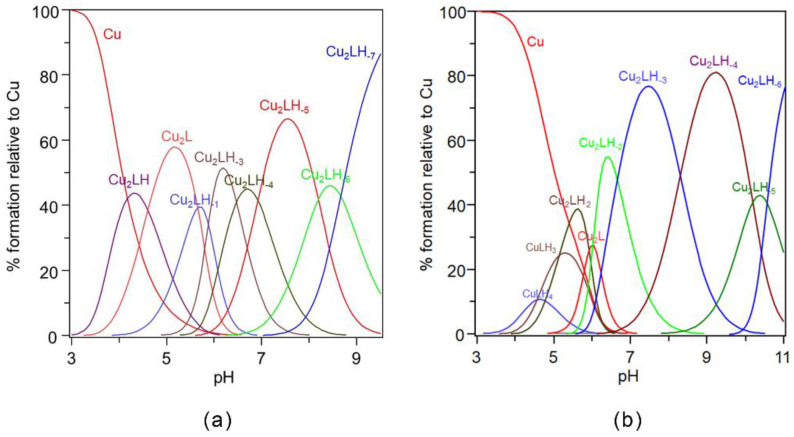
Species distribution of copper(II) complexes with: (**a**) Ang1-17; and (**b**) AcAng1-17. [L] = 1 × 10^−3^ M; metal-to-ligand molar ratio of 2:1.

The stability constant values obtained are reported in Table 1 and the corresponding distribution diagram is shown in Figure 3a.

The Ang1-17 peptide has five protonation constants ([LH_5_], i.e., two carboxylic side chains, two imidazole rings and the terminal amino moiety), whereas the stability constant values of deprotonation of the Tyr and Lys side chains for this ligand were not determined due to precipitation phenomena, as previously reported [45].

Starting from pH = 3, the [Cu_2_LH] complex species is formed. The number of released protons and the stability constant value suggest that each copper ion may be bound to one imidazole and one carboxylate group. It is also possible that an isomer in which N-terminal amino group is bound to copper with one imidazole still protonated. However, spectroscopic data cannot be determined for [Cu_2_LH] species due to the presence of free copper ions and other metal complexes species. Therefore, it is not possible to make specific assertions about the main metal anchoring sites at low pH values.

Increasing the pH, the [Cu_2_L] complex species is formed. Potentiometric data (log K_210_ = log β_211_ − log β_210_ = 4.60) indicate the simultaneous involvement of two imidazole nitrogen atoms in copper binding to determine a (2N_Im,_ O_COO−_) coordination environment for one metal ion whereas the second Cu(II) ion may be bound through NH_2_ terminal group (NH_2_, O_COO−_) [47]. The UV-vis spectroscopic parameters confirm this coordination mode (see Table 1, Figure S1).

Increasing the pH, the [Cu_2_LH_−1_] is formed. Due to the superimposition of the other complex species, the UV-vis parameters could not be determined for this complex species. In the CD spectrum recorded at pH = 5.8 (Figure S2), a N^−^ → Cu(II) CT band centered at 338 nm (overlapped with the N_Im_ → Cu(II) CT band) is present that, combined with the log K value determined by potentiometric titrations, is a concrete clue to the involvement of a deprotonated nitrogen atom in this complex species (log K_21-1_ = log β_210_ − log β_21-1_ = 5.65) [48,49].

The ESR spectrum run on Cu(II)-Ang1-17 at pH = 5.5, shows the patterns of at least two coordination modes, beside the signal due to due the copper(II) hexaaquo ion. Comparing spectra at different pH values, it is clear that the spectrum run at pH = 5.5 also contains a contribution deriving from the features of the species found at a pH value of 6.0 (which is, in turn, very similar to that found at pH = 7.4). To ease the computation, the spectral contribution of the ESR spectrum at pH = 6.0 was subtracted from that taken at pH = 5.5 and the resulting spectrum was simulated (Figure 2e). A satisfactory simulation was achieved by using the following Hamiltonian parameters: g_||_ = 2.245, A_||_ = 186 × 10^−4^ cm^−1^ and g_||_ = 2.295, A_||_ = 176 × 10^−4^ cm^−1^, in agreement with the environments identified by potentiometric and UV-vis data.

On raising the pH value, a cooperative deprotonation of two nitrogen atoms occurred, hence the [Cu_2_LH_−3_] species was formed and reached its maximum percentage of formation at pH = 6.4. The two Cu(II) ions have very similar coordination environments, as evidenced by the symmetric and quite sharp UV-vis band centered at 602 nm. Two 3N1O coordination modes can be hypothesized for the two Cu(II) binding site: in the first environment NH_2_, N^−^, N_Im_, O_COO−_ is present at the peptide N-terminus, whereas a N_Im_,2N^−^ coordination mode involves the second Cu(II) ion. The deprotonation of the two coordinating nitrogen atoms is confirmed by their averaged log K value (5.88 × 2) as well as by the intensity increase in the N^−^ → Cu(II) CT band at 338 nm observed in the CD spectrum recorded at pH = 6.3.

Regarding the [Cu_2_LH_−4_] species, another peptide nitrogen atom deprotonation occurred, as evidenced by its log K value. Unfortunately, an unequivocal assignment of the residue involved in the nitrogen deprotonation was not possible due to the similarity of the two Cu(II) coordination sites.

The main complex species present at physiological pH is the [Cu_2_LH_−5_] species, in which both copper(II) ions are coordinated to the peptide by four nitrogen atoms. The first coordination mode involves NH_2_, 2N^−^, N_Im_ donor atoms, whereas the second Cu(II) ion is bound to the peptide by an imidazole nitrogen atom and three deprotonated amide nitrogen atoms, i.e., N_im_, 3N^−^. A very broad UV-vis band was recorded for this complex, resulting from two partially superimposed gaussian peaks centered at λ_max_ of 585 nm and 535 nm and characterized by a comparable intensity, due to the two CuN_4_ chromophores (i.e., NH_2_, 2N^−^, N_Im_ and N_im_, 3N^−^, respectively).

In the pH range 6–8 only an ESR pattern was recorded. The averaged parameters drawn directly from these ESR spectra are g_||_ = 2.218, A_||_ = 191 × 10^−4^ cm^−1^ (Figure 2f). These spectra were satisfactorily simulated by using two sets of Hamiltonian parameters, i.e., g_||_ = 2.228, A_||_ = 184 × 10^−4^ cm^−1^ and g_||_ = 2.208, A_||_ = 187 × 10^−4^ cm^−1^. Additionally, the ESR spectrum run at pH = 8.5 (not shown) shows a broadening on the parallel features due to the presence of similar coordination environments, and their averaged Hamiltonian parameters are g_||_ = 2.209, A_||_ = 194 × 10^−4^ cm^−1^.

Finally, upon increasing the pH above the physiological value, another two complex species were formed, i.e., [Cu_2_LH_−6_] and [Cu_2_LH_−7_]. A 4N coordination mode (N_Im_, 3N^−^ and NH_2_, 3N^−^, respectively) for the two copper(II) centers occurs, as demonstrated by the presence of a UV-vis band centered at 532 nm. Further support for this hypothesis comes from the presence in the CD spectrum of the CT bands at 270 nm (N_im_ π_2_ → Cu^2+^), at 301 nm (NH_2_ → Cu^2+^) and at 343 nm (N_im_ → Cu^2+^ overlapped with N^−^ → Cu^2+^). Only above pH = 8.5 sharp parallel features appeared in the ESR spectra with parameters approximately keeping the same values shown at physiological pH (Figure 2g), i.e., g_||_ = 2.196, A_||_ = 205 × 10^−4^ cm^−1^.

### 2.3. ESR Study on Cu(II)-AcAng1-17 System (M/L 1:1)

The ESR spectrum of Cu(II)-AcAng1-17 with equimolar amounts of metal and ligand at a pH of around 4 reveals the features of two complex species as well as those belonging to copper(II) hexaaquo ion (data not shown). The Hamiltonian parameters of the two complex species are g_||_ = 2.332, A_||_ = 158 × 10^−4^ cm^−1^ and g_||_ = 2.295, A_||_ = 176 × 10^−4^ cm^−1^, attributable to [CuLH_4_] and [CuLH_3_] species, are compatible with N_Im_, O_COO−_ and 2N_Im_, O_COO−_ coordination modes, respectively.

**Figure 4:**
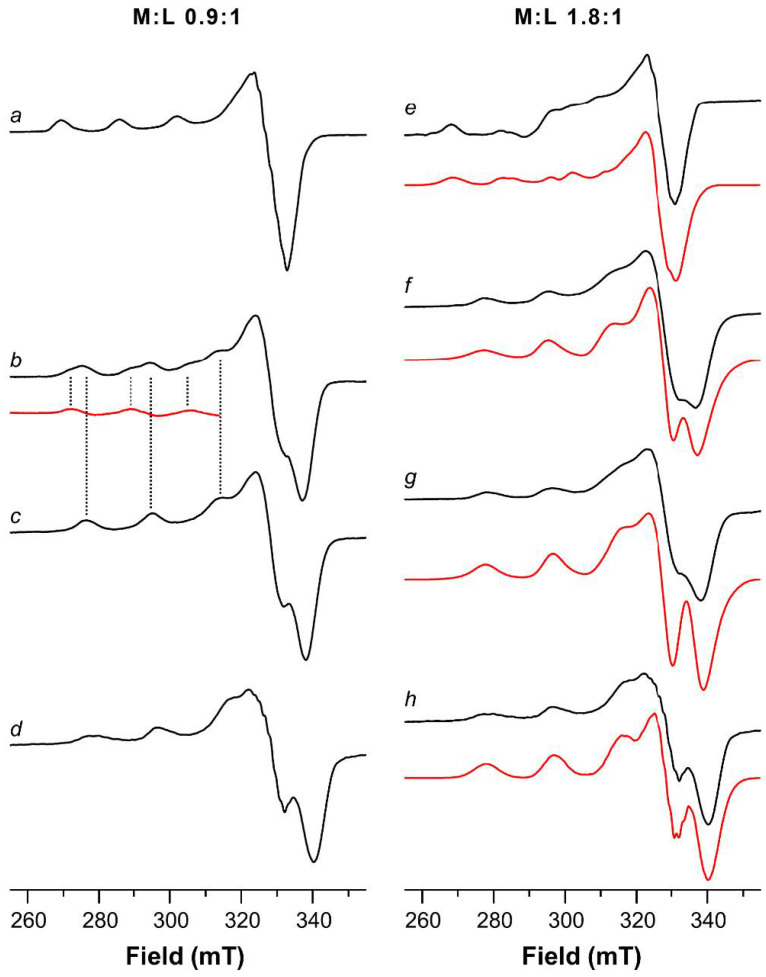
ESR spectra at 150 K of ^63^Cu(II)-AcAng1-17 system (**a**–**d**, M:L 0.9:1) and ^63^Cu(II)-AcAng1-17 system (**e**–**h**, M:L 1.8:1) in aqueous solution as function of the pH: (**a**) 5.0; (**b**) 6.0 (the red trace represents the parallel features of the spectrum of trace at pH 6.0 minus trace at pH 6.9); (**c**) 6.9; (**d**) 10.8; (**e**) 4.3 (subtracted from [^63^Cu(H_2_O)_6_]^2+^ features); (**f**) 6.4; (**g**) 7.6; (**h**) 10.8. Simulated spectra on the right panel are shown by the red line.

Only a species having the latter set of parameters is observed at pH = 5 (Figure 4a), i.e., the [CuLH_3_] species.

At pH = 6 the patterns belonging to two different species are clearly visible in the ESR spectrum (Figure 4b) characterized by g_||_ = 2.266, A_||_ = 180 × 10^−4^ cm^−1^ and g_||_ = 2.212 and A_||_ = 196 × 10^−4^ cm^−1^. The former set is assigned to [CuLH_2_] species, whereas the latter to the [CuLH] species (Figure 4c), the ESR spectrum of which begins to be present at pH = 6 and is the only detectable until pH = 8. The hypothesized coordination modes for [CuLH_2_] and [CuLH] species are 2N_Im_, N^−^, and 2N_Im_, 2N^−^, respectively.

On raising the pH value, last amide nitrogen is deprotonated and coordinates the Cu(II) ion, the ESR spectra being characterized by g_||_ = 2.199 and A_||_ = 205 × 10^−4^ cm^−1^ (Figure 4d). These parameters are compatible with a N_Im_, 3N^−^ coordination mode.

### 2.4. Potentiometric and ESR Studies on Cu(II)-AcAng1-17 System (M/L 2:1)

Despite the lack of the amino group, the peptide AcAng1-17 still possesses two anchoring sites for Cu(II) ion, i.e., the two His residues. With the aim of testing the ability to form dicopper(II) species, two equivalents of the Cu(II) ion were added to a solution of the AcAng1-17 peptide.

The AcAng1-17 peptide is also able to form up to a 2:1 metal-to-ligand ratio complex species with Cu(II) ions. The stability constant values obtained are reported in Table 2 and the corresponding distribution diagrams in Figure 3b, respectively.

Differently from the case of the non-acetylated Ang1–17 peptide, all protonation stability constants of AcAng1–17 peptide were determined (two carboxylic side chains, two imidazole rings and one Tyr and two Lys side chains), as previously reported [45]. At acidic pH values, two complex species, ([CuLH_4_] and [CuLH_3_]), each binding only one Cu(II) ion, were found.

In the 4.3–5.6 pH range, the ESR spectra revealed, besides some residual Cu(II) hexaaquo ion, the presence of two distinct patterns of signals. The parameters obtained by simulation of the spectrum (Figure 4e, red trace) are g_||_ = 2.290, A_||_ = 170 × 10^−4^ cm^−1^ and g_||_ = 2.332, A_||_ = 158 × 10^−4^ cm^−1^ and can be assigned to [CuLH_3_] and [CuLH_4_], respectively, with copper coordination modes N_Im_, N^−^, O_COO−_ and N_Im_, O_COO−_.

Around pH values of 5.5, [Cu_2_LH_2_] complex species is formed. The deconvoluted UV-vis spectrum for this complex species showed a very broad band resulting from the overlap of two distinct bands (Figure S3). The determined λ_max_ values (705 nm and 635 nm) are indicative of two Cu(II) ions bound to the peptide via two different coordination modes. The UV-vis band centered at 705 nm is typical of a 1N1O N_Im_, O_COO−_ coordination mode, whereas the band centered at 635 nm corresponds to a 2N1O N_Im_, N^−^, O_COO−_ environment, as found in previous works [50,51]. In the deconvoluted CD spectrum, a band with a maximum centered at 334 nm is found, which is an overlap between the CT bands related to the N_im_ → Cu(II) and the N^−^ (amide) → Cu(II) CT transitions, respectively (Figure S4).

Then, the occurrence of a cooperative deprotonation with the consequent formation of the [Cu_2_L] complex species was found only by potentiometric technique. Its averaged log K value is very similar to a typical value found for the deprotonation of two amide atoms [52]. Unfortunately, a spectroscopic characterization was not feasible.

Increasing the pH, another cooperative deprotonation of two nitrogen atoms occurred with the formation of the [Cu_2_LH_−2_] complex species, as determined by potentiometric data, which are in a good agreement with those found in the literature. The deconvoluted UV-vis spectrum for this complex species (λ_max_ = 565 nm) is a linear combination of two single bands. The first band is centered at 535 nm, which is in a good agreement with a 4N (N_Im_, 3N^−^) coordination mode for a Cu(II) ion [53]. Instead, the second band is centered at 590 nm, hence supporting the presence of a 3N (N_Im_, 2N^−^) coordination mode for the other metal ion [50].

In the CD spectrum a band with a maximum centered at 326 nm was found. Such a broad band is a combination of the CT bands relative to a N_Im_ → Cu(II) transition and to a N^−^ → Cu(II) transition. In addition, the band centered at 260 nm corresponds to a N_Im_ π_2_ → Cu(II) charge transfer transitions. Finally, the bands centered at 486 nm, 546 nm, 609 nm and 681 nm refer to the d-d transitions of both coordination sites [54].

The ESR spectrum run at pH value of 6.4 was quite broad (Figure 4f); a simulation was performed considering two species and the following parameters were found g_||_ = 2.220, A_||_ = 184 × 10^−4^ cm^−1^ and g_||_ = 2.200, A_||_ = 190 × 10^−4^ cm^−1^. These parameters agree with the coordination modes hypothesized for Cu_2_LH_-2_ species using UV-vis spectroscopy and CD measurements.

At physiological pH, the main complex species is [Cu_2_LH_−3_]. The calculated log K for this step is indicative of another deprotonation of nitrogen atom, which led to the formation of two equivalent complexation sites in which every single Cu(II) ion is coordinated to the peptide by a 4N (N_Im_, 3N^−^) donor set. This coordination mode is confirmed by the g_||_ and A_||_ values extracted from the ESR spectrum recorded at pH = 7.6 (Figure 4g, Table 1). The UV-vis band for this complex species is narrow, very symmetric and its λ_max_ is centered at 532 nm. This value is in a good agreement with those found for similar peptide systems having the same coordination environment [53].

In the CD spectrum calculated for this complex species, the same CT and d-d bands observed for the previous species were found, but in this case the N^−^ → Cu(II) and the N_Im_ → Cu(II) CT transitions are distinct, with the first being centered at 320 nm and the second at 360 nm. These values are compatible with the coordination environment proposed above [47].

Upon increasing the pH above 7, another three deprotonation steps were found without significant variations in UV-vis, CD and ESR spectra. (Figure 4h, Table 2 and Table 3). Potentiometric data indicate that these deprotonation steps come from the OH and NH_2_ moieties of one Tyr and two Lys side chain residues, respectively [50].

### 2.5. Voltammetric Study on Cu(II)-Ang1-17 System (M/L 1:1)

The electrochemical data were collected at different pH values between 3.5 and 11.0, covering the range of copper complex species formation. The formal redox potentials together with the ESR parameters are reported in Table 3.

**Figure 5:**
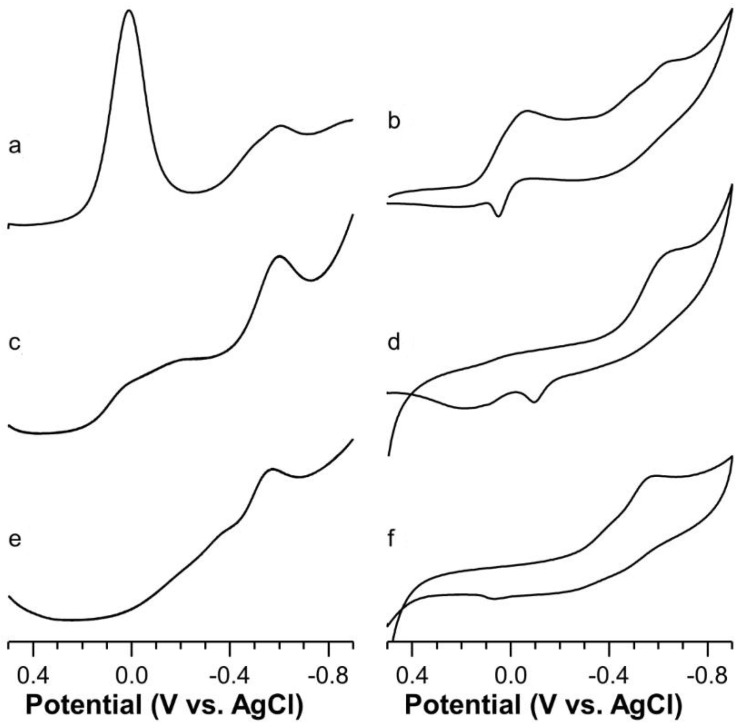
Square wave (left) and cyclic (right) voltammograms of Cu(II)-Ang1-17 system (C_L_ = 5 × 10^−4^ M, M:L 0.9:1, KNO_3_ 0.1 M) in aqueous solution as function of the pH: (**a**,**b**) 4.5; (**c**,**d**) 7.3; (**e**,**f**) 11.0.

Square wave voltammogram (SWV) carried out on a solution of Cu-Ang1-17 at pH = 4.5 (M/L 1:1, I = 0.1 M KNO_3_) showed a peak at −0.610 V (beside the presence of the Cu(II) hexaaquo ion peak) with a shoulder at −0.495 V (Figure 5a). The deconvolution of the voltammetric curve by using PeakFit 4.0 program (Jandel) gave as result two peaks centered at −0.592 V and −0.509 V, thus giving evidence of two kinds of coordination polyhedra. The quite negative value of the former potential value suggests the formation of a distorted octahedral Cu(II) coordination polyhedron, attributable to the NH_2_, N_Im_, N^−^, O_COO-_ donor set. In fact, despite of the involvement of only two nitrogen atoms in the Cu(II) coordination, the strong basicity of the amino moiety must be considered. In addition, the coordination of a deprotonated amide and a negative charged carboxylate oxygen atom increases the charge density of the Cu(II) atom, hence hindering the reduction process. All these factors concur to strengthen the ligand field, which means that a quite large absolute value of A_||_ (185 × 10^−4^ cm^−1^) and a quite negative value for the reduction process (−0.592 V) of Cu(II) is observed. The other deconvoluted peak (centered at −0.515) V can be assigned to 2N_Im_, O_COO-_ donor set, and the formal redox potential value is indicative of a distorted tetragonal chromophore [55].

The cyclic voltammogram (CV) showed two cathodic peaks (Figure 5b); the first can be assigned to Cu(II) hexaaquo ion reduction at about −0.045 V, whereas the other peak, at −0.625 V with a shoulder at −0.494 V, is due to the reduction processes of the two Cu(II) coordination species. The re-oxidation anodic peaks of these latter processes are not clearly separated but coalesce into a single broad peak having a mean potential of −0.446 V. Overall, these processes can be considered quasi-reversible.

On raising the pH toward neutrality, the potential peak intensities due to Cu(II) hexaaquo ion electrochemical processes progressively decrease and disappear, whereas those of the two above described complex species increase progressively and show broad peaks due to different individual contributions.

At physiological pH values, and up to pH = 8, SWV traces present a well pronounced peak centered at −0.585 V (Figure 5c) characteristic of strong ligand field due to a slightly distorted CuN4 chromophore. This hypothesis agrees with ESR data assigned to [CuLH_−1_] species.

CV scans at pH = 7.3 showed a cathodic peak at −0.615 V (Figure 5d) which is associated with the anodic reoxidation at −0.490 V. The peak-to-peak separation is about 130 mV and the i_a_/i_c_ ratio is about 0.5, which means that this process can also be considered quasi-reversible.

From pH 8 to pH 10 and higher, the SWV peak centered at −0.585 V progressively moves towards the E°_f_ value of −0.550 V at pH = 11 (Figure 5e). This potential shift corroborates the distortion of the Cu(II) coordination plane already seen in the ESR measurements. The CVs show a cathodic peak at −0.555 V (Figure 5f), but the corresponding anodic peak is nearly absent. This behavior may account for a rearrangement of the coordination environment due to the different demand of Cu(I), upon reduction of Cu(II), i.e., diagonal or trigonal geometries.

### 2.6. Voltammetric Study on Cu(II)-AcAng1-17 System (M/L 1:1)

**Figure 6:**
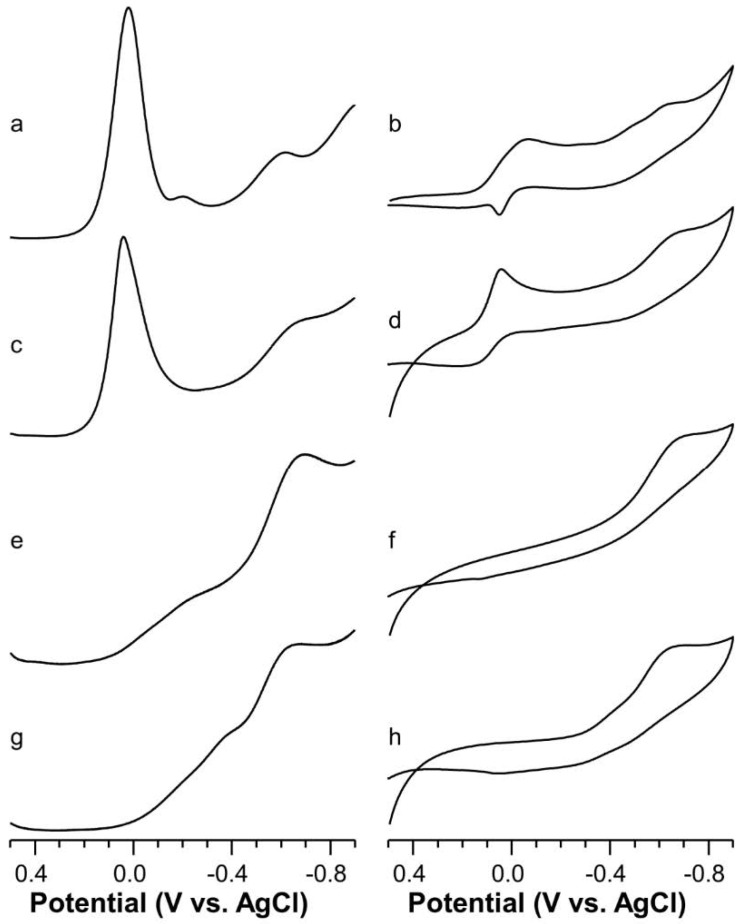
Square wave (left) and cyclic (right) voltammograms of Cu(II)-AcAng1-17 system (C_L_ = 5 × 10^−4^ M, M:L 0.9:1, KNO_3_ 0.1 M) in aqueous solution as function of the pH: (**a**,**b**) 4.5; (**c**,**d**) 6.0; (**e**,**f**) 8.0; (**g**,**h**) 11.0.

A square wave voltammogram (SWV) carried out on a solution of Cu-AcAng1-17 at pH = 4.5 (M/L = 1, I = 0.1 M KNO_3_) evidenced a peak at about −0.585 V and a smaller peak (−0.209 V) at the foot of the Cu(II) hexaaquo ion (Figure 6a). The small peak may be attributed to the coordination of an imidazole moiety to the Cu(II) with a partial contribution of the carboxylate (about 20% protonated, pK = 3.90) [45].

The deconvolution of the broad peak resulted in two peaks: −0.515 V and −0.597 V. The value of −0.515V is that found for the 2N_Im_, O_COO−_ donor set, whereas the more negative value may be attributed to the coordination by at least two histidines. These processes could be related to different isomers previously found for [CuLH_4_] species [45]. The formal potential of −0.597 is nearly the same as that attributed to the 2N_Im_, N^−^ coordination environment at higher pH values (Table 3).

CV showed a cathodic peak due to the Cu(II) reduction, followed by two cathodic reduction peaks at −0.237 and −0.600 V (Figure 6b). Only a small but broad anodic peak can be associated with this latter reduction.

The SWV run at pH = 6 showed a broad peak positioned at −0.642 (Figure 6c) and a sharp peak at about +0.040 V. The intensity of this latter peak, together with the triangular shape of the associate cathodic peak in the CV experiment (Figure 6d), suggests the occurrence of an adsorption process in addition to the reduction process of residual uncomplexed Cu(II) ion (as expect from distribution species diagram). The deconvolution of the broad peak resulted in two peaks centered at −0.594 V and −0.662 V. The first value was assigned to 2N_Im_, N^−^ coordination whereas the more negative value can be ascribed to 4N coordination, i.e., 2N_Im_, 2N^−^ in which a further deprotonated amide nitrogen atom takes the place of carboxylate oxygen atom.

In the pH range from 7 to 8, a peak is only detected in the SWV traces. The associated E°_f_ value is −0.656 V (Figure 6e) is well-suited to a planar Cu(II) coordination environment formed by four nitrogen donor atoms, which is in good agreement with the ESR spectra. In addition, the CV voltammograms exhibit a single reduction (cathodic) peak at −0.660 V and its associated anodic wave is at −0.477 V (Figure 6f).

Finally, at the most alkaline pH values the E°_f_ value shifts towards −0.615 V (Figure 6g). This value is still in agreement with a four-nitrogen coordination environment, but the positive shift indicates a distortion of the plane.

### 2.7. Voltammetric Study on Cu(II) with Ang1-17 or AcAng1-17 Systems (M/L 2:1)

The addition of two equivalents of Cu(II) ion to both peptides resulted in the formation of different coordination environments because of the distribution of the metal ions among different anchoring points.

**Figure 7:**
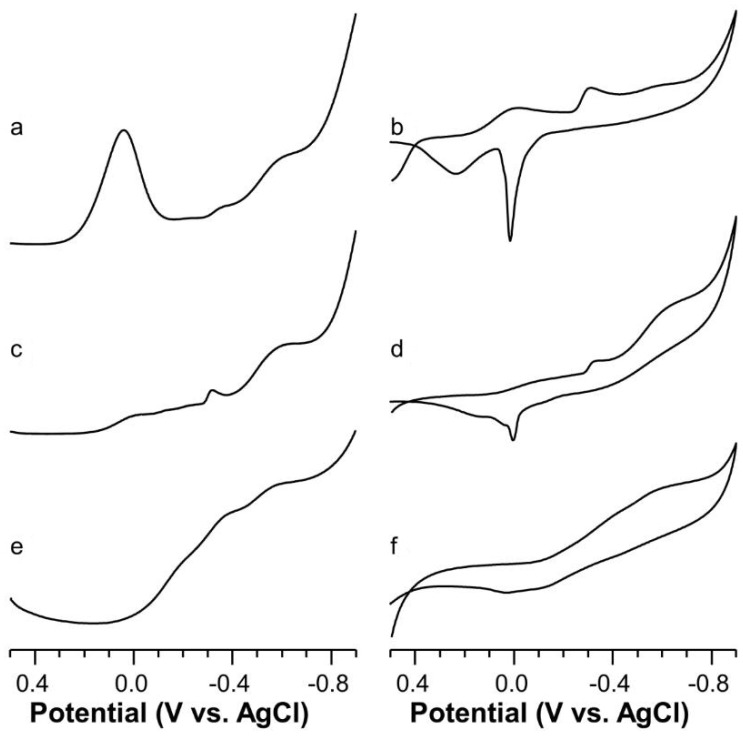
Square wave (left) and cyclic (right) voltammograms of Cu(II)-Ang1-17 system (C_L_ = 5 × 10^−4^ M, M:L 1.8:1, KNO_3_ 0.1 M) in aqueous solution as function of the pH: (**a**,**b**) 6.0; (**c**,**d**) 7.3; (**e**,**f**) 11.0.

The SWV voltammograms run on Cu(II)-Ang1-17 system (M/L 2:1) at pH = 6 show two main peaks (Figure 7a). The first peak may be due to the presence of residual Cu(II) uncomplexed ions, whereas the second fairly broad peak is placed at −0.571 V. The deconvolution of the broad peak at −0.571 V unveils the presence of two peaks having E°_f_ values of −0.520 V and −0.590 V, due to the reduction of two different CuN_2_O_2_ chromophores: the first is formed by 2N_im_, O_COO−_, H_2_O donor set, whereas the most negative peak potential can be ascribed to NH_2_, N^−^, O_COO−_, H_2_O.

At almost physiological value, the SWV trace presents a main broad peak centered at −0.585 V (Figure 7c). The outcome of the deconvolution of the latter wave gave two peaks centered at −0.534 V and −0.618 V, which suggest the presence of distorted N_Im_, 3N^−^ and NH_2_, 2N^−^, N_Im_ donor sets, respectively. This last coordination scenario is evident at higher pH values and the E°_f_ value moves towards the −0.571 V (Figure 7e). The more positive E°_f_ value found at high pH confirms the distortion of the four nitrogen donor atoms as also inferred by the high values of absorptivity found at high pH values. The formation of more distorted structural isomers cannot be ruled out as a small shoulder can be seen at −0.355 V on the SWV trace.

Concerning the AcAng1-17 peptide, the amino group acetylation hinders its coordination ability and the number of preferred anchoring points for Cu(II) reduces essentially to the two histidine residues. As a consequence, the number of species present in the solution drastically decreases.

**Figure 8:**
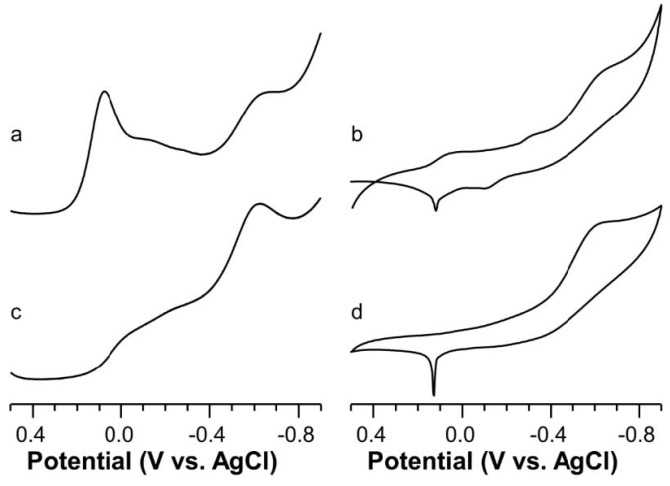
Square wave (left) and cyclic (right) voltammograms of Cu(II)-AcAng1-17 system (C_L_ = 5 × 10^−4^ M, M:L 1.8:1, KNO_3_ 0.1 M) in aqueous solution as function of the pH: (**a**,**b**) 6.4; (**c**,**d**) 7.6.

At pH = 6.4, the SWV trace present minor redox active species and some residual free copper ions and a broad peak at −0.610 V (Figure 8a). At this pH value both [Cu_2_LH_−1_] and [Cu_2_LH_−3_] species are present, and their formal potentials values were estimated by deconvolution, the outcomes of which were −0.565 V and −0.631 V, respectively.

A quite similar peak centered at −0.600 V (Figure 8c) was obtained at pH = 7.6 where only [Cu_2_LH_−3_] species is present with two very similar coordination environments showing different degrees of distortion from planarity, i.e., E°_f_ values of −0.582 V and −0.631 V, obtained by peak deconvolution.

### 2.8. Peptides Effect on Intracellular Copper Contents in SH-SY5Y Cell Line

Neuroblastoma cell lines SH-SY5Y were stained with copper sensor1 (CS1), an intracellular fluorescent probe of monovalent copper [34]. The red emission of the CS1 fluorophore component due to the BODIPY group, is enhanced when the Cu^+^ chelator moiety binds to the intracellular copper. Confocal microscopy imaging was used to scrutinize the cells and investigate if the whole ANG protein (wild-type and recombinant isoforms), as well as Ang1-17 and AcAng1-17 peptides, could influence copper cellular content. The experiments were performed in the presence or absence of copper ions.

**Figure 9:**
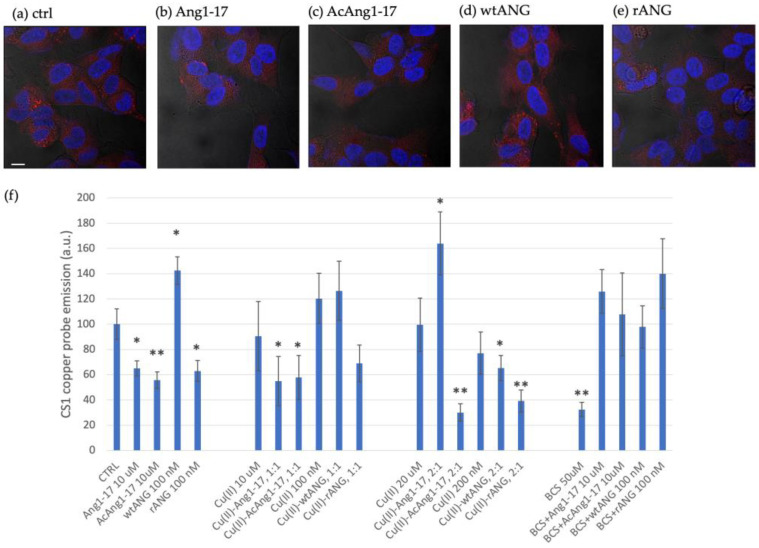
Representative confocal microscopy merged micrographs of fluorescence channels (in blue, nuclear staining, λ_ex/em_ = 405/425–450 nm; in red, CS1-copper probe, λ_ex/em_ = 543/550–600 nm) and optical bright field (in grey) of SH-SY5Y cells untreated (**a**, negative control) and after 90 min of treatment with: (**b**) 10 µM Ang1-17, (**c**) 10 µM AcAng1-17, (**d**) 100 nM wtANG, and (**e**) 100 nM rANG. Scale bar = 10 μm. In (**f**) is shown the quantitative analysis of CS1-copper probe emission intensity for the cells treated as follows (four blocks from left to right): in basal medium, untreated or 10 µM peptide-/100 nM protein-treated cells; in CuSO_4_-supplemented medium, equimolar copper/peptide or copper/protein ratio; in CuSO_4_-supplemented medium, 2:1 copper/peptide or copper/protein molar ratio; in copper deprived medium by pretreatment with BCS extracellular copper chelator, in the absence or presence of 10 µM peptides/100 nM proteins. Bars represent means ± SEM of at least 3 experiments; (*) = *p* < 0.05, (**) = *p* < 0.01, vs. CTRL (untreated cells in basal medium) (Student’s *t*-test).

The histogram in Figure 9 shows the response to the Cu^+^ reporter for the different treatment conditions.

It is important to note that in the series of cellular treatments in basal medium, incubation with both free peptides, Ang1-17 or AcAng1-17, induced a decrease in red fluorescence inside cells. These data suggest that both peptides are able to bind the copper ions present in the basal medium, which can reach micromolar concentration [56], and can shift the balance of copper from inside the cell to outside. On the other hand, the incubation with wtANG showed a strong increase in intracellular copper fluorescence, while treatment with rANG induced a significant decrease in intracellular copper compared to the negative control of untreated cells.

In a parallel series of cell treatments, in order to have control over the very variable trace copper in the medium, typically ranging from submicromolar to a few micromolar units [56], we used the BCS extracellular copper chelator [57,58]. The histograms in Figure 9f show that, besides the reference pretreatment with 50 µM BCS, no significant changes in the intracellular copper could be detected for any of the treatments, either with the peptides or the proteins.

Interestingly, in the copper-supplemented medium, after cell incubation with equimolar copper/peptide or copper/protein concentrations, only in the case of the treatments with the peptides was a decrease in CS1 intensity in the cytosol still detected. Moreover, in the case of an excess of copper, at 2:1 molar ratio, while the amount of copper inside the cell increased upon the treatment with Ang1-17, a significant decrease was detected after incubation with AcAng1-17, wtANG or rANG.

## 3. Discussion

The ability to chelate copper(II) ions and the characterization of the species formed with Ang1-17 and AcAng1-17 have been investigated by using M/L 1:1 and 2:1. Potentiometric, spectroscopic and voltammetric techniques are of great help to understand which species form as a function of pH and which donor atoms are involved in each complex species. In particular, ESR spectroscopy also allows the characterization of copper complexes geometry that determines the redox potential of the Cu(II)/Cu(I) pair, a critical parameter in determining the efficiency of most biochemical reactions involving copper [59,60]. In a previous work, we determined the species formed by the peptides Ang(1-17) and AcAng1-17 with Cu(II) ion at M/L 1:1 by means of potentiometric studies [45]. Specifically, we found that the Ang1-17 peptide started to complex Cu(II) forming [CuLH] species, where the amino group acts as main anchoring site via its nitrogen atom. The coordination environment of the chromophore is completed by a deprotonated amide nitrogen atom, the Asp carboxylic oxygen and a water molecule.

Another important fact to note is that a second possible concurrent anchoring site would be the imidazole nitrogen of His8 which, together with the histidine amide nitrogen, the Asp15 carboxylic oxygen and a water molecule, form a very similar chromophore. Potentiometry does not allow us to highlight the presence of structural isomers, since it measures macroconstants, whereas ESR and voltammetric measurements may show the presence of isomers if they have a different coordination environment.

The ESR spectra run on a frozen solution of Cu(II)-Ang1-17 system in the 3.5–3.9 pH range showed the presence of several patterns due to different species and residual Cu(II) hexaaquo ion. The first set of parameters (g_||_ = 2.335, A_||_ = 146 × 10^−4^ cm^−1^) can be attributed to the CuN_2_O_2_ chromophore containing the amino group, an imidazole nitrogen and a carboxylic oxygen atoms in a distorted octahedral polyhedron [61]. The second set of parameters (g_||_ = 2.295, A_||_ = 176 × 10^−4^ cm^−1^) can be assigned to a chromophore formed by two imidazole nitrogen atoms and a deprotonated carboxylate (Asp), as previously reported [62,63], despite of the lack of a close sequence proximity of the two histidine residues.

On raising the pH value to 4.5, the main ESR active species is characterized by g_||_ = 2.250 and A_||_ = 185 × 10^−4^ cm^−1^. This species can be assigned to [CuL] species, where the metal is coordinated by NH_2_, N_im_, N^−^, O_COO−_ chromophore, i.e., the amino group, an imidazole ring nitrogen atom, a deprotonated amide and a carboxylic oxygen atoms [45,64], hence forming a distorted CuN_3_O chromophore. This coordination features are confirmed by the presence of a strong negative peak at −0.592 V in the voltammetric curve. The formation of this species is also supported by the coordination environment of the [CuLH_−1_] species formed in the 5.3–8.2 pH range. At pH values close to neutral, such a complex is fully formed by the deprotonation of an additional amide nitrogen atom replacing the carboxylic oxygen atom, i.e., NH_2_, N_Im_, 2N^−^. This CuN_4_ chromophore has a slightly distorted octahedral geometry.

Notably, for a four-nitrogen in-plane a higher A_||_ value is expected associated with a more negative value of E°_f_. Instead, this deprotonation caused only a modest increase in the A_||_ value, indicating that a significant distortion of the coordination plane occurred. This is supported by a well pronounced peak centered at −0.585 V in the SWV trace, confirming the presence of a slightly distorted CuN_4_ chromophore [45].

Above pH = 9.2 only a single species characterized by g_||_ = 2.195, A_||_ = 203 × 10^−4^ cm^−1^ was detected by ESR spectrometry. These magnetic parameters are consistent with the presence of [CuLH_-2_] species which formed after deprotonation of a third amide nitrogen atom. This latter donor atom replaces the amino moiety so as the chromophore is always a distorted CuN_4_ but a stronger ligand field is experienced by the metal ion, as confirmed by the increase in the hyperfine coupling constant and the decrease in the value of d-d band. Nevertheless, a higher A_||_ value would also be expected in this case; hence some distortion of the coordination plane persists, as also indicated by the ε increase from 106 to 130 M^−1^ cm^−1^ on going from pH 8.5 to 11.0 [45]. The value of −0.550 V obtained by SWV is more positive with respect to previous species (−0.585 V) and is in line with the trend shown by ESR spectroscopy.

The formation of isomers observed when one equivalent of copper(II) ion is added to the Ang1-17 peptide gave us a clue about the its ability to accept up to two equivalents of the metal.

No attempt to characterize the [Cu_2_LH] complex species using the ESR data were performed because of the large amount of copper(II) hexaaquo ion that is present at very acidic pH values. Additionally, the [Cu_2_L] complex species was not clearly recorded at low temperature (150 K). Actually, at the pH value of 5.5 an ESR pattern attributable to the subsequent [Cu_2_LH_−1_] species is probably found because the low temperature favors its formation over the [Cu_2_L] species. The Hamiltonian parameters obtained for the [Cu_2_LH_−1_] species (obtained from simulation, i.e., g_||_ = 2.245, A_||_ = 186 × 10^−4^ cm^−1^ and g_||_ = 2.295, A_||_ = 176 × 10^−4^ cm^−1^) can be assigned to two coordination environments formed by NH_2_, N^−^, O_COO−_ and 2N_Im_, O_COO−_ donor sets, respectively, analogously to those obtained for [CuLH] isomers (at metal-to-ligand ratio of 1:1). The presence of two metal centers with two different coordination environments is confirmed by the deconvolution of the broad peak at −0.571 V detected in the SWV trace. There are two peaks with E°_f_ values of −0.590 V assigned to a Cu(NH_2_, N^−^, O_COO−_) chromophore, and of −0.515 V assigned to Cu(2N_Im_, O_COO−_) chromophore.

The main complex species [Cu_2_LH_−3_], [Cu_2_LH_−4_] and [Cu_2_LH_−5_] are present in the 6–8 pH range. The corresponding ESR spectra showed slightly broadened features and the parameters directly extracted are g_||_ = 2.218, A_||_ = 191 × 10^−4^ cm^−1^. The ESR spectra were satisfactorily simulated by using two sets of Hamiltonian parameters, i.e., g_||_ = 2.228, A_||_ = 184 × 10^−4^ cm^−1^ and g_||_ = 2.208, A_||_ = 187 × 10^−4^ cm^−1^. Notably, the g_||_ value of 2.218, drawn directly from the spectra, is the average of the g-values found in the spectral simulation. A variety of coordination environments was also found for copper(II) complexes with 1-16 fragments of β-amyloid [35]. In particular, the murine Ac-1-16M fragment forms a species having g_||_ = 2.233, A_||_ = 187 × 10^−4^ cm^−1^, which were attributed to N_Im_, 2N^−^, H_2_O donor set [61], and these values are close to the first couple of parameters obtained by simulation. The human 1-16 fragment forms instead a species characterized by g_||_ = 2.203, A_||_ = 187 × 10^−4^ cm^−1^ [64], which were assigned to a NH_2_, 2N^−^, N_Im_ donor set and are nearly identical to the couple of computed values (although a NH_2_, 3N^−^ chromophore cannot be completely ruled out). All these parameters are nearly equivalent to the obtained computed values, hence confirming the formation of two non-equivalent (3N and 4N) coordination modes. It can be reasonably inferred that at low temperature the [Cu_2_LH_−4_] species, which possess N_Im_, 3N^−^ and NH_2_, N_Im_, N^−^ donor sets, may prevail.

In fact, the assignment to the [Cu_2_LH_−3_] complex species, which has two nearly equivalent coordination environments, can be discarded. In addition, the [Cu_2_LH_−5_] species is characterized by two four-nitrogen coordination environments which should exhibit two g_||_ values close to 2.20 and lower, as well as larger A_||_ values, as observed at pH values higher than 8.0. At physiological pH the deconvolution of the peak at −0.585 V gave two peaks centered at −0.534 V and −0.618 V, which confirm the contemporary presence of N_Im_, 2N^−^ and NH_2_, 2N^−^, N_Im_ donor sets, respectively.

The ESR spectrum run at pH = 8.5 shows a broadening of the parallel features due to the presence of similar coordination environments. The averaged Hamiltonian parameters taken from this ESR spectrum are g_||_ = 2.209, A_||_ = 194 × 10^−4^ cm^−1^ which may be tentatively assigned to the two nearly equivalent copper(II) centers of [Cu_2_LH_−5_] species.

Above pH = 9 the ESR spectra remain unvaried, with sharp parallel features and Hamiltonian parameters keeping approximately the same values recorded at pH = 8.5, i.e., g_||_ = 2.196, A_||_ = 205 × 10^−4^ cm^−1^, which are assigned to two nearly equivalent coordination environments, N_Im_, 3N^−^ and NH_2_, 3N^−^, having a four-nitrogen distorted coordination plane. Very similar values were also found for dinuclear Cu(II) complexes with (1-2,7-21)NPG having such coordination environments [54]. The E°_f_ value at −0.571 V value, increasing the pH agrees with the presence of four nitrogen atoms in a slight distorted geometry around copper.

The spectra obtained in the 10–11 pH range, as well as their Hamiltonian parameters, are practically unvaried. At these pH values the deprotonation of lysine and tyrosine residues occurs, but these groups are not involved in the Cu(II) coordination.

The AcAng1-17 peptide has two histidine residues only as main anchoring points for copper(II) ion, being the amino group acetylated. The acetylation led to a reduction of the dinuclear species. Interestingly, when one Cu(II) equivalent is added to the peptide solution at pH 4, one or both histidine residues can chelate. In fact, the ESR spectrum showed features (beside those of copper(II) hexaaquo ion) belonging to two complex species. A first set of parameters (g_||_ = 2.332, A_||_ = 158 × 10^−4^ cm^−1^) can be attributed to the initial Cu(II) anchoring by an histidine imidazole moiety assisted by a carboxylate oxygen donor atom, which is consistent with the [CuLH_4_] species [65]. A SWV peak having a E°_f_ value of −0.209 V was assigned to redox process of this coordination environment. Instead, the second species (g_||_ = 2.295, A_||_ = 176 × 10^−4^ cm^−1^) is due to the Cu(II) coordination by 2N_Im_, O_COO−_, H_2_O already observed for Cu(II)-Ang1-17 at acidic pH, which is the only species recorded by ESR at pH = 5, i.e., the [CuLH_3_] species. Such coordination mode is confirmed by the peak −0.515 V observed in SWV, analogous to that observed for analogous Cu-Ang1-17 complex.

Two ESR active species were found at pH = 6. The first species has g_||_ = 2.266, A_||_ = 180 × 10^−4^ cm^−1^ and despite the lowering of the g_||_ value due to an additional amide donor atom, the hyperfine coupling constant remains almost unvaried. This may be due to a distorted coordination geometry in which the amide nitrogen atom replaces the carboxylate oxygen, i.e., 2N_Im_, N^−^ equatorial donor set. Such a coordination hypothesis for [CuLH_2_] species is supported by similar spectroscopic data found for Cu-Ac-GHHPHG-NH_2_ system, and describing the same donor set [66]. The other species, which begins to be present at pH = 6, and is the only detectable one until pH = 8, is the [CuLH] species. This species presents Hamiltonian parameters which are typical for a four-nitrogen planar coordination environment (g_||_ = 2.212, A_||_ = 196 × 10^−4^ cm^−1^). Its formation is due to the deprotonation of an additional amide nitrogen atom which contributes to the formation of the (2N_Im_, 2N^−^) equatorial donor set. The presence of two complex species at pH = 6 is further confirmed by the deconvolution of the broad peak in SWV, resulting in two peaks centered at −0.594 V and −0.662 V, ascribed to redox processes of Cu(2N_Im_, N^−^, O_COO−_), Cu(2N_Im_, 2N^−^), respectively. The latter process occurring in the 6.9–8.1 pH range showed practically the same formal potential value.

On raising the pH value, the last amide nitrogen is deprotonated and coordinates Cu(II) by taking the place of an imidazole histidine nitrogen atom, hence forming a N_Im_, 3N^−^ donor set. The coordination of the third amide nitrogen atom causes, as expected, the raising of the hyperfine coupling constant and the lowering of the g_||_, (i.e., g_||_ = 2.199 and A_||_ = 205 × 10^−4^ cm^−1^) which have been observed for similar coordination modes. The E°_f_ value of −0.580 V measured in the SWV confirms the presence of a four nitrogen atoms in a distorted arrangement around copper(II).

With the aim of testing the ability to form dicopper(II) species, two equivalents of Cu(II) were added to a solution of AcAng1-17 peptide. At acidic pH values (4.3–5.6 pH range) ESR features belonging to two different species were detected. The simulation of the ESR spectrum gave as a result g_||_ = 2.332, A_||_ = 158 × 10^−4^ cm^−1^ and g_||_ = 2.290, A_||_ = 170 × 10^−4^ cm^−1^. The former parameters can be assigned to N_Im_, O_COO−_ coordination environment already described for the [CuLH_4_] species [45]. The [CuLH_3_] species is instead described by the latter parameters, which are well suited for N_Im_, N^−^, O_COO−_ coordination environment [51].

The ESR spectrum run at pH value of 6.4, apparently disclosing features due to a single species. In fact, a certain degree of broadening indicates that quite similar but different coordination environments are formed. A simulation of the ESR spectrum gave g_||_ = 2.220, A_||_ = 184 × 10^−4^ cm^−1^ and g_||_ = 2.200, A_||_ = 190 × 10^−4^ cm^−1^, which can be attributed to the (N_Im_, 2N^−^) and (N_Im_, 3N^−^) coordination environments, respectively. Such coordination modes can be attributed to [Cu_2_LH_−2_] complex species. This is confirmed by the presence of two cathodic peaks at −0.565 V and −0.631 V assigned to (N_Im_, 2N^−^) and (N_Im_, 3N^−^) coordination mode, respectively.

Starting from about pH = 7 both coordination environments have (N_Im_, 3N^−^) donor sets and the ESR spectra showed few variations in the g-values and their corresponding A_||_-values, indicating a different degree of distortion from the planarity between the two coordination sites. These deviations from planarity are also reflected by the recorded formal potential, which moves towards a more positive value.

The overall experimental findings indicate that both peptides can bind up to two copper ions in excess of metal concentration. The direct comparison between the stability constant values cannot provide the indication on which peptide binds copper ions more strongly since they form complex species with different deprotonation states.

Being aware of the competition of different ligands in the extracellular space, the ability to bind copper, the coordination environment and geometry can make these angiogenin-derived peptides potential players in the variation of copper levels between outside and inside the cell.

The intracellular copper level was studied by means of LSM using a fluorescent probe able to detect the Cu(I) present inside the cell. Copper at a concentration of 10 μM or 20 μM increases the amount of copper inside cells, as expected. On the other hand, the addition of free peptide decreases the intracellular copper level, suggesting that both Ang1-17 and Ac Ang1-17 bind copper present in the medium, shifting the balance on both sides of the membrane, so as to induce the cell to release the metal in the extracellular space.

The contemporary addition of an equimolar amount of copper and peptide at 10 μM does not reverse the trend to decrease the presence of copper inside cell driven by peptides. By using a 2:1 metal-to-ligand molar ratio, an increase in intracellular copper is observed for peptide Ang1-17, indicating that the peptide can actually act as ionophore or copper shuttle in cases of an excess of copper. This drastic change can be caused by the different copper complex speciation and different coordination environments around the metal when the peptide Ang1-17 binds two copper ions compared to species formed at 1:1 ratio. Remarkably, in the same conditions the peptide AcAng1-17 further decreases the intracellular copper content, maintaining a chelating behavior. Therefore, the environmental conditions can determine a different behavior by the same ligand.

The addition of BCS, a copper chelator, nullified the effect of peptides and copper cells’ content was equal to control.

The effect of the whole protein on intracellular copper levels was also determined. The addition of wtANG induced a strong increase in intracellular copper compared to control. Additionally, for the protein, the addition of BCS inhibits the increase in intracellular ion induced by wtANG, providing indirect evidence that the protein favors the copper cellular uptake.

It can be hypothesized that the protein favors the entry of copper directly or through the activation of other signaling pathways. It is noteworthy that the addition of the recombinant protein, rANG, decreases the copper signal inside cells, as observed for the addition of peptides Ang1-17 and AcAng1-17. It is of note that the only difference between wt-Ang and rANG is the presence of a Met(-1) with a free amino group in the recombinant isoform [44]. The two proteins display the same RNase activity and have the same cellular binding site, showing differences only in presence of copper ions [44]. The decrease in intracellular copper induced by rANG may be ascribed to a direct binding of protein with metal ions, so the rANG may have a metal-chelating activity, as supposed for both Ang1-17 and AcAng1-17 peptides. This is further confirmed by the decrease in copper uptake when the rANG is added together with copper in both 1:1 and 2:1 metal to protein molar ratio; it must be considered that the recombinant protein can bind up two metal ions per molecule [44]. It is important to note that the presence of the thioether group of methionine in rANG could provide an additional donor atom that would also favor copper(I) binding. The obtained data for rANG are similar to those for peptides that do not contain methionine, suggesting the absence of a thioether interaction with the metal ion. Differently, the wtANG can bind only one copper ion and the addition of one or two equivalent show a level of copper comparable to free copper addition at the same concentration. The addition of copper together with wtANG decreases the copper uptake compared to the protein alone.

A hypothesis to rationalize these data is that wtANG can favor cellular uptake through direct binding but in defect of copper and not in presence of equimolar or excess equivalents of metal. At equimolar amounts of copper and wtANG, an equilibrium is achieved whereby the protein no longer carries copper inside. However, these data demonstrate that angiogenin can modulate the cellular uptake of copper. Considering that copper regulates angiogenin expression in endothelial cells, our findings support a strict correlation in protein and metal activity.

It is worth emphasizing the different effect induced by recombinant that limits the uptake of copper present in the culture medium. Frequently, studies on the activity of angiogenin are carried out using the more available recombinant form. This is justified considering that rANG displays the same activities as that wild type ANG normally present in human plasma. Here, we demonstrate that the wild type protein must necessarily be used in the presence of copper to avoid an incorrect conclusion.

The interaction between angiogenin and copper represents a potential target in many pathologies where a dyshomeostasis of copper and an abnormal protein expression are observed, as in tumors.

Peptide fragments belonging to the whole protein able to partially mimic some activities of protein may potentially be used as drugs in these pathologies.

## 4. Materials and Methods

### 4.1. Chemicals

The peptide QDNSRYTHFLTQHYDAK-NH_2_ (Ang1-17) and the acetylated analogous peptide Ac-QDNSRYTHFLTQHYDAK-NH_2_ (AcAng1-17) were supplied by Caslo Aps, Lyngby, Denmark. All other chemicals, of the highest available grade, were purchased from Sigma-Aldrich (Munich, Germany) and used without further purification. For the cellular experiments, Dulbecco’s modified eagle medium (DMEM)-F12, penicillin-streptomycin solution, L-glutamine, fetal bovine serum (FBS), Dulbecco’s phosphate-buffered saline (PBS) and paraformaldehyde were purchased from Sigma-Aldrich (St. Louis, MO, USA).

### 4.2. Expression and Purification of Angiogenin

The human ANG expression was carried out according to Holloway et al.’s procedure [67]. Briefly, the *E. coli* (BL21(DE3)) expression strain was cultured at 37 °C under shaking (180 rpm) in 5 mL of terrific broth (12 g peptone, 24 g granulated yeast extract, 4 mL glycerol 87%, 900 mL of MilliQ water) supplemented with ampicillin (100 μg/mL). The volume of the bacterial culture was inoculated in 1000 mL of fresh broth, after 24 h. The ANG expression was induced by the addition of 1 mM isopropyl β-D-1-thiogalactopyranoside (IPTG). Subsequently, the cell culture was harvested by centrifugation (4000 RCF for 15 min at 4 °C, JLA 8100) and cells were lysed by means of lysis buffer (50 mM Tris-HCl, 2 mM EDTA, pH = 8), using the high-pressure homogenizer (Emulsiflex) and a sonication step (Qsonica Sonicator Q700, Newtown, CT, USA). Afterwards, the lysate was centrifuged (20,000 RCF for 40 min at 4 °C, JA25.50) and the pellet was re-suspended in 25 mL of lysis buffer supplemented with 1% (*v*/*v*) Triton X-100. Sonication and centrifugation steps were repeated twice and the final pellet was dissolved in 30 mL of denaturation buffer (0.24 M guanidine hydrochloride (GdnHCl), 100 mM Tris-HCl, 1 mM ethylenediaminetetraacetic acid (EDTA), 4 mM NaCl, 0.4 mM 1,4-dithiothreitol (DTT)).

The recombinant angiogenin obtained (r-Ang) encompasses a methionine as first residue, Met(-1) and was refolded from inclusion bodies according to the procedure described by Jang et al. [68]. The purification was performed by means of a cation exchange chromatography performed on an automated chromatographic workstation (Akta prime, GE Healthcare, Chicago, Illinois, USA) equipped with a 15 × 1.6 cm column packed with SP Sepharose Fast Flow (GE Healthcare, Chicago, Illinois, USA). The protein was eluted with 25 mM Tris-HCl, 1 M NaCl (pH = 8.0) buffer solution.

It is necessary remove the first methionine residue in order to obtain the angiogenin protein with the native sequence, here named wtANG.

The rANG was incubated with 1 nM *Aeromonas aminopeptidase*, an enzyme that specifically cut the first methionine, at the concentration of 1 × 10^−5^ M in 200 mM potassium phosphate buffer (pH = 7.2) (overnight at 37 °C under gentle shaking) [10.1016/0003-2697(88)90569-6]. After the remotion of Met(-1), the N-terminal glutamine residue (Glu1) spontaneously cyclizes to the pyroglutamate residue in order to obtain the wtANG. The reaction mixture was purified by dialysis (Spectra/por MWCO 6–8000 Da), which replaces PBS with 25 mM Tris-HCl (pH 7.4) buffer solution, followed by cation-exchange chromatography.

### 4.3. Electron Spin Resonance Spectroscopy (ESR)

A Bruker Elexsys E500 CW-EPR spectrometer driven by a PC running the XEpr software and equipped with a Super-X microwave bridge operating at 9.3–9.5 GHz and a SHQE cavity was used throughout this work. All ESR spectra of frozen solution of Cu(II) complexes were recorded at 150 K by means of a ER4131VT variable temperature apparatus. In order to increase spectral resolution, a small amount of methanol (not exceeding 10%) was added to the Cu(II) complex aqueous solutions, after adjusting the pH to the desired value, to obtain a good quality glass upon freezing [69]. The ESR magnetic parameters g_||_ and A_||_ were extracted from the 2nd and the 3rd line to remove second order effects [70]. Some experimental spectra were simulated by the program Monoclin [71], which allows researchers to distinguish more species simultaneously present.

### 4.4. Potentiometric Titrations

Potentiometric titrations were performed on a Titrando 905 automatic titrator (Switzerland) using a combined glass-Ag/AgCl electrode (Metrohm, Switzerland) The titration cell (2.5 mL) was thermostated at 298.0 ± 0.2 K, and all solutions were kept under an atmosphere of argon. KOH solutions (0.1 M) were added through a Hamilton burette equipped with 1 cm^3^ syringe. The ionic strength of all solutions was adjusted to 0.10 M (KNO_3_). In order to determine the stability constants, solutions of the ligands with copper(II) ion were titrated using 0.1 M potassium hydroxide. Ligand concentration ranged from 1.0 to 1.5 × 10^−3^ M. A minimum of three independent runs were performed to determine the copper(II) complexation constants [72].

Metal-to-ligand ratio of 2:1 was employed. The initial pH was always adjusted to 2.4. To avoid systematic errors and verify reproducibility, the electromotive force (EMF) values of each experiment were taken at different time intervals.

To obtain complexation constants, the potentiometric data were refined using Hyperquad [73]. The species distribution as a function of the pH was obtained using the computer program Hyss [74].

### 4.5. Ultraviolet-Visible (UV-vis) Measurements

UV-vis spectra were recorded at 25 °C using an Agilent 8453 (Agilent Technologies, Santa Clara, CA, USA) or a Jasco V-670 (Jasco, Easton, MD, USA) spectrophotometer. The concentrations of the peptides and copper(II) ion used to record absorption spectra were the same as those for the potentiometric titrations.

Combined spectroscopic and potentiometric metal-complex titrations were performed in a 3 mL quartz cuvette with a 1 cm path length to obtain the spectrum in the visible region at each pH value simultaneously. These experiments were replicated at least three times for each copper–peptide system. Spectroscopic data were processed by means of Hyperquad program [60]. The deconvolution carried out by using Peakfit 4.0.

### 4.6. Circular Dichroism (CD) Measurements

CD spectra were obtained at 25 °C under a constant flow of nitrogen on a Jasco model 810 spectropolarimeter (Jasco, Easton, MD, USA) at a scan rate of 50 nm min^−1^ and a resolution of 0.1 nm, the path length being 1 cm, in the 280–800 nm range. The spectra were recorded as an average of either 3 or 5 scans. Calibration of the instrument was performed with a 0.06% aqueous solution of ammonium camphorsulfonate. The CD spectra of the copper(II) complexes on varying the solution pH were obtained in both the 190–250 and 250–800 nm wavelength regions. All the solutions were freshly prepared using double distilled water. The copper(II) ion and peptide concentrations used for the acquisition of the CD spectra in the visible region were identical to those used in the potentiometric titrations. The results are reported as ∆ε (molar dichroic coefficient) in M^−1^ cm^−1^.

### 4.7. Voltammetric Study

Cyclic voltammograms (CV) of the Cu(II) complexes in solution (5 × 10^−4^ M, 0.1 M KNO_3_ as ground electrolyte) were recorded by means of a Metrohm Autolab PGSTAT 128N potentiostat-galvanostat driven by a standard PC. These solutions were analyzed by using a Metrohm glass cell with a three-electrode assembly: a working glassy carbon electrode (2 mm diameter), a glassy carbon rod as auxiliary electrode and a Ag/AgCl reference electrode. All electrodes and parts were manufactured by Metrohm. The cell also hosted a combined 3-mm Metrohm Biotrode micro pH glass electrode which was connected with a titrator Metrohm Titrando 905 dispensing KOH 0.1 M directly in the cell. The PGSTAT 128N and Titrando 905 are coupled by programming the communications of the driving software (Tiamo 2.4 and Nova 1.11.2) released by Metrohm. Complex solutions were degassed by using ultrapure argon. Electrochemical measurements were generally acquired by sweeping the potential from +0.500 to −0.900 V. The square wave voltammetry (SWV) experiments were carried out a 15 Hz frequency with a 25 mV applied pulse value. Cyclic voltammetry (CV) measurements were carried out at 25 °C with sweep rate of 50 mV s^−1^ in the same potential range of SWV experiments. All reported potentials are referred to Ag/AgCl reference electrode, +0.212 V vs. Normal Hydrogen Electrode (NHE), unless otherwise stated. The Ag/AgCl electrode potential was checked by using methylviologen redox couple (MV^2+^/MV^+^), −0.446 V vs. NHE [75].

### 4.8. Cell Cultures

Human neuroblastoma (SH-SY5Y) cells were cultured in Dulbecco’s modified eagle medium (DMEM)-F12 medium supplemented with 10% (*v*/*v*) FBS, 2 mM L-glutamine, 100 U penicillin/0.1 mg/mL streptomycin. Cells were grown in tissue-culture treated Corning^®^ flasks (Sigma-Aldrich, St. Louis, MO, USA) under a humidified atmosphere of air/CO_2_ (95:5) at 37 °C in an incubator (Heraeus Hera Cell 150C incubator).

### 4.9. Confocal Microscopy Analysis

Confocal imaging microscopy was performed with a FV1000 confocal laser scanning microscope (LSM), furnished with diode UV (405 nm, 50 mW), multiline Argon (457 nm, 488 nm, 515 nm, total 30 mW), HeNe(G) (543 nm, 1 mW) and HeNe(R) (633 nm, 1 mW) lasers. An oil immersion objective (60xO PLAPO) and spectral filtering systems were used. The detector gain was fixed at a constant value and images were collected, in sequential mode, randomly all through the area of the dish. All images were deconvolved with Huygens Essential software (Scientific Volume Imaging, The Netherlands).

To perform the experiment, SH-SY5Y cells were seeded on glass bottomed dishes (WillCo-dish^®^, Willco Wells, B.V.) with 12 mm of glass diameter, at a density of 30 × 10^3^ cells per dish, in DMEM-F12 medium with 10% (*v*/*v*) FBS. After 24 h, medium was changed with DMEM-F12 supplemented with 1% (*v*/*v*) FBS and cells were treated for 90 min with: 0.1 µM, 0.2 µM, 10 µM and 20 µM; 10 µM CuSO_4_; 10 µM Ang1-17 and AcAng1-17 in the absence and in the presence of CuSO_4_ at the concentrations of 10 µM and 20 µM, corresponding to a Cu/peptide molar ratio of 1 and 2, respectively; 0.1 µM wtANG and rANG, in the absence and in the presence of CuSO_4_ at the concentrations of 0.1 µM and 0.2 µM, corresponding to a Cu/protein molar ratio of 1 and 2, respectively.

Following the incubation time, cells were stained with nuclear dye Hoechst33342 (0.25 µg/mL) (Thermo-Fisher Scientific) and 1 µM CS1-copper probe and then fixed with high purity 2% (*w*/*v*) paraformaldehyde in PBS.

## Figures and Tables

**Table 1:** Log β and log K values obtained from the titration of the Cu–Ang1-17 system (2:1), by varying the solution pH in the range 3–9.5.

Species [Cu_p_L_q_H_r_]	log β *	log K	UV (λ_max_, ε_max_) (nm, M^−1^ cm^−1^)	CD (λ, ∆ε) (nm, M^−1^ cm^−1^)
211	17.28 (1)	-	-	-
210	12.67 (2)	4.60	650 (110)	266 (+0.25), 299 (−1.15), 602 (+0.25)
21-1	7.02 (2)	5.65	-	-
21-3	−4.74 (2)	5.88 × 2	602 (227)	260 (+7.43), 303 (−1.45), 342 (+0.45), 532 (+0.33), 625 (−0.33)
21-4	−11.21 (3)	6.47	-	-
21-5	−18.13 (3)	6.92	See text	261 (+8.90), 301 (−0.73), 321 (+0.35), 350 (−0.35), 491 (−1.00), 669 (+0.78)
21-6	−26.33 (3)	8.19	-	-
21-7	−34.99 (3)	8.66	532 (380)	270 (+3.31), 302 (+0.97), 343 (−0.98), 514 (−0.93), 661 (+1.06)

* 3σ in parentheses.

**Table 2:** Log β and log K values obtained from the titration of the Cu-AcAng1-17 system (2:1), by varying the solution pH in the range 3–11.

Species [Cu_p_L_q_H_r_]	log β *	log K	UV (λ_max_, ε_max_) (nm, M^−1^ cm^−1^)	CD (λ, ∆ε) (nm, M^−1^ cm^−1^)
114	40.38 (1)	-	- **	- **
113	35.82 (1)	4.56	650 (60) **	258 (2.52); 331 (0.30); 598 (−0.24) **
212	33.50 (2)	-	See text	334 (+0.04); 528 (+0.03),
21-0	21.95 (2)	(5.77 × 2)	-	-
21-2	9.56 (3)	(6.19 × 2)	See text	260 (+6.69); 326 (+0.47); 486 (−0.33); 546 (+0.28); 609 (−0.19); 681 (+0.30)
21-3	2.94 (3)	6.62	532 (250)	262 (+7.30); 320 (+0.70), 360 (−0.27), 495 (−1.08), 560 (+0.32), 665 (+0.90)
21-4	−5.36 (6)	8.30	528 (242)	264 (+7.95); 347 (−1.08); 492 (−1.12); 650 (+1.35)
21-5	−15.55 (8)	10.19	-	-
21-6	−26.10 (5)	10.65	528 (230)	265 (+8.05); 347 (−0.66); 501 (−2.06); 646 (+1.30)

* 3σ in parentheses; ** Reference [45].

**Table 3:** ESR parallel Hamiltonian parameters and formal redox potentials of copper(II) with Ang1-17 and AcAng1-17 at different metal-to-ligand ratios.

***Cu(II)-Ang1-17 1:1***				
**pH**	**g_||_**	**A_||_**	**Donor Set**	**E°_f_ (SWV)**
3.9	2.424	124	6H_2_O	
	2.250	185	NH_2_, N^−^, O_COO−_	
	2.295	176	2N_im_, O_COO−_	
	2.335	146	NH_2_, N_im_, O_COO−_	
4.5–5.3	2.295	176	2N_im_, O_COO−_	−0.515
	2.250	185	NH_2_, N_Im_, N^−^, O_COO−_	−0.592
5.3–8.2	2.224	186	NH_2_, N_Im_, 2N^−^	−0.585
9.2	2.195	203	N_im_, 3N^−^	−0.550
10.5	2.195	201	N_im_, 3N^−^	−0.550
***Cu(II)-Ang1-17 2:1***				
5.2–6.2	2.245	186	NH_2_, N^−^, O_COO−_	−0.590
	2.295	176	2N_im_, O_COO−_	−0.515
6.2–7.9	2.228	184	N_Im_, 3N^−^	−0.534
	2.208	187	NH_2_, Nim, 2N^−^	−0.618
8.5	2.209	194		
9.2–11	2.196	205	N_im_, 3N^−^NH_2_, 3N^−^	−0.571
***Cu(II)-AcAng1-17 1:1***				
3.8	2.424	124	6H_2_O	
	2.332	158	N_im_, O_COO−_	−0.209
	2.295	176	2N_im_, O_COO−_	−0.515
5.0	2.295	176	2N_im_, O_COO−_	
6.0	2.266	180	2N_im_, N^−^	−0.594
	2.212	196	2N_im_, 2N^−^	−0.662
6.9–8.1	2.212	196	2N_im_, 2N^−^	−0.656
9.3	2.206	200	N_im_, 3N^−^	−0.580
10.8	2.199	205	N_im_, 3N^−^	−0.615
***Cu(II)-AcAng1-17 2:1***				
4.3–5.6	2.332	158	N_im_, O_COO−_	
	2.290	170	N_im_, N^−^, O_COO−_	
6.4	2.220	184	N_im_, 2N^−^	−0.565
	2.200	190	N_im_, 3N^−^	−0.631
7.6	2.205	190	N_im_, 3N^−^	−0.631
	2.195	198	N_im_, 3N^−^	−0.582
8.5–9.5	2.190	198	N_im_, 3N^−^	
10.8	2.195	205	N_im_, 3N^−^	
	2.185	195	N_im_, 3N^−^	

## Supplementary Materials

The following are available online at https://www.mdpi.com/article/10.3390/ijms22179530/s1.
