# Supplementary material for: Peptides Derived from Angiogenin Regulate Cellular Copper Uptake
Source: Int J Mol Sci. 2021 Sep 2;22(17):9530. doi: 10.3390/ijms22179530 (PMC8430698; doi:10.3390/ijms22179530)
Supplement: Supplementary file 1 [file ijms-22-09530-s001.zip › ijms-1246191-SI.pdf]

Supplementary Materials

Peptides Derived from Angiogenin Regulate Cellular Copper Uptake

Giovanni Tabbì [1], Lorena Maria Cucci [2], Calogero Pinzino [3], Alessia Munzone [4], Tiziano Marzo [5], Silvia Pizzanelli [3,*], Cristina Satriano [2,*], Antonio Magrì [1,*] and Diego La Mendola [5,*]

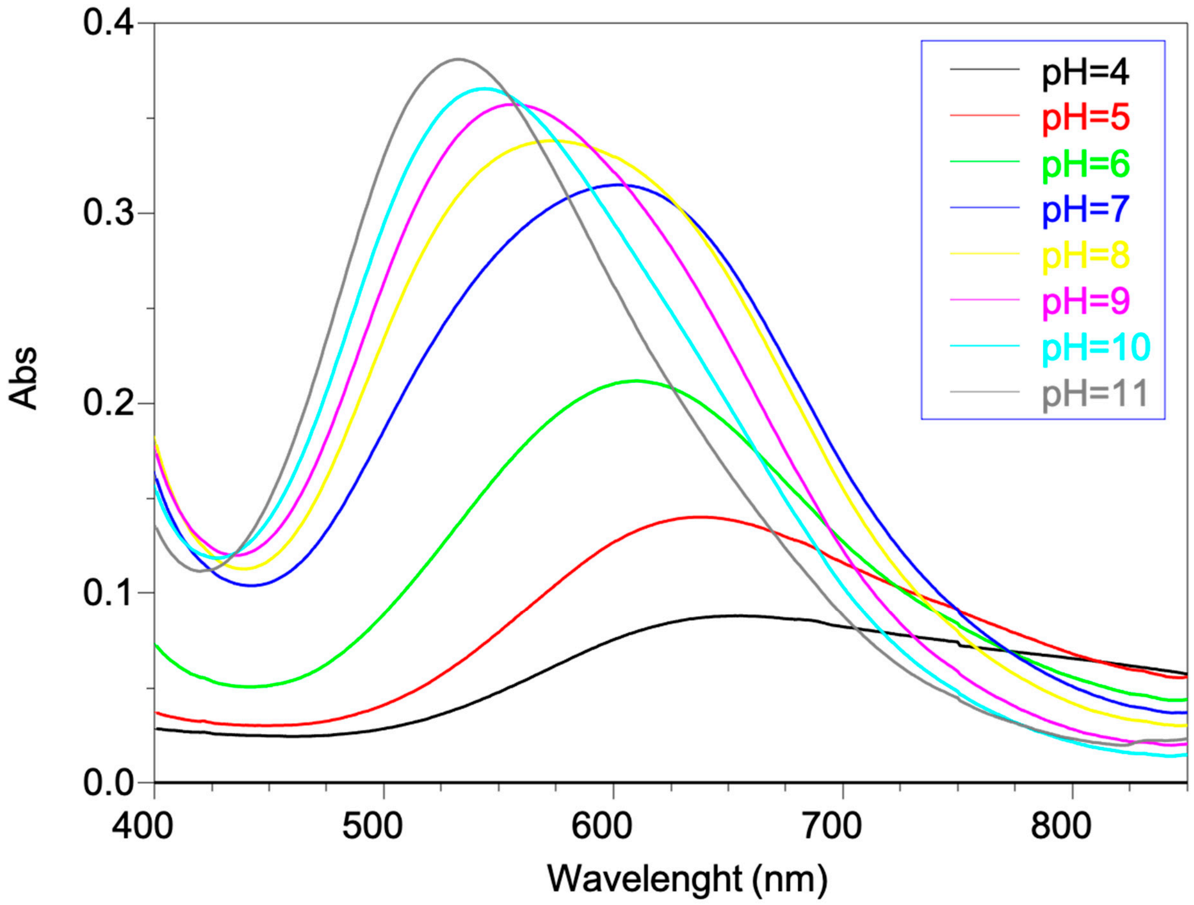

Figure S1. UV-vis spectra of Cu(II)-Ang1-17 at different pH values. [L] = $1x10^{-3}$ M, metal to ligand molar ratio 2:1.

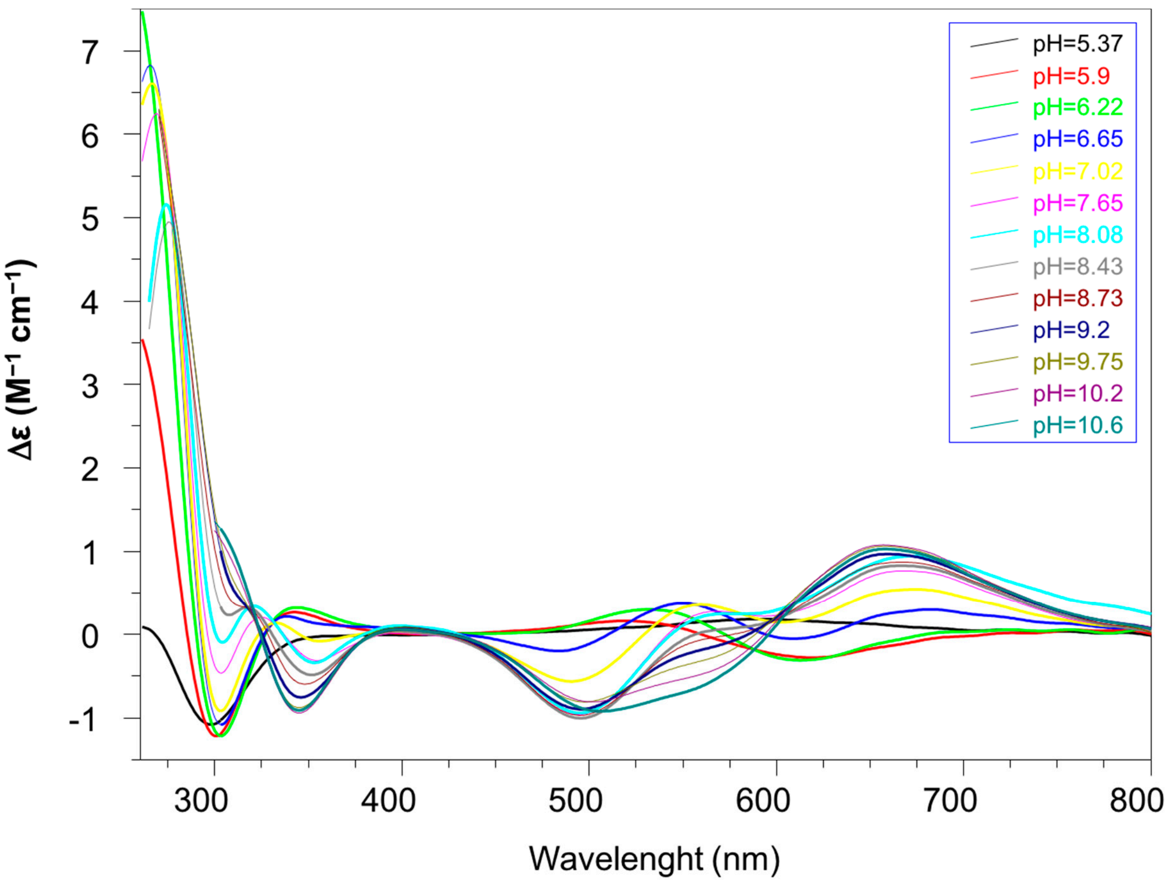

Figure S2. CD spectra of Cu(II)-Ang1-17 at different pH values. [L] = $1x10^{-3}$ M, metal to ligand molar ratio 2:1.

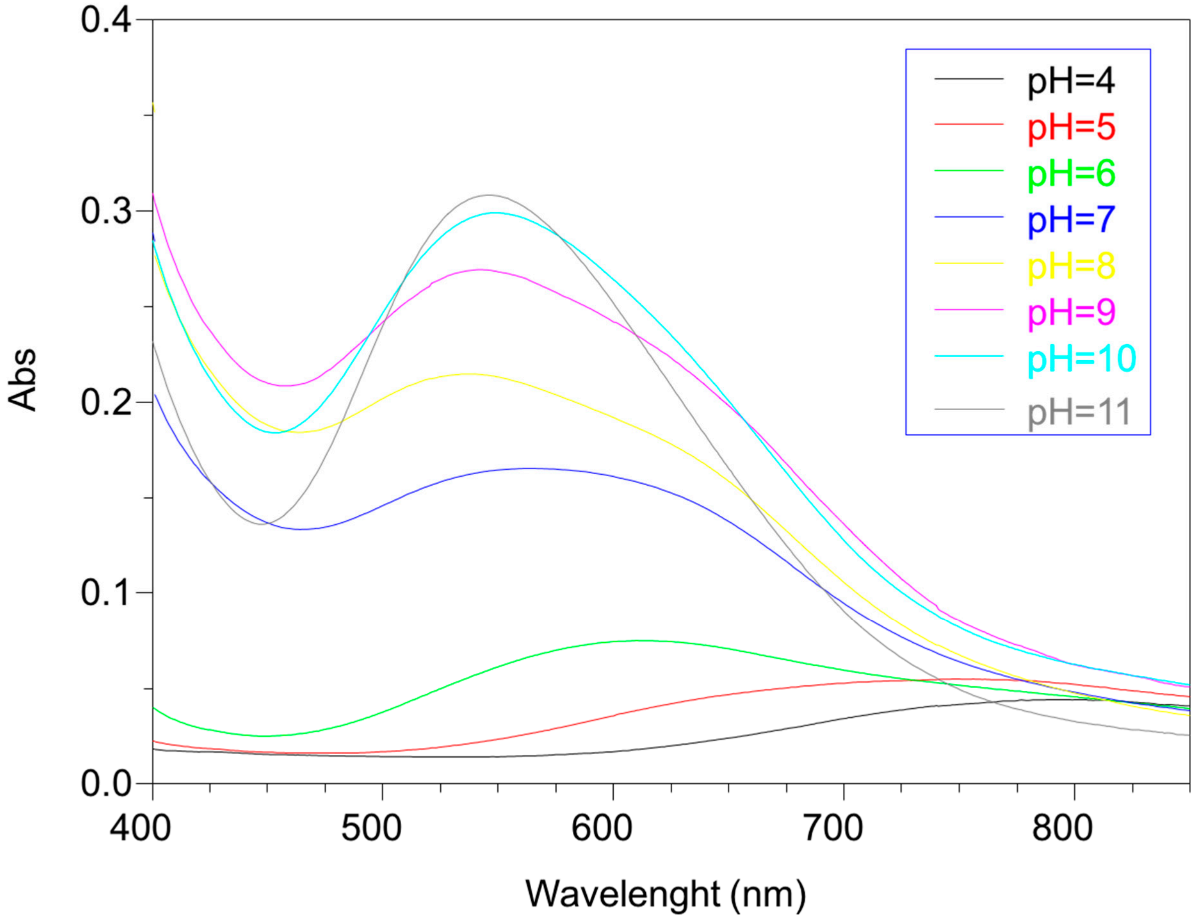

Figure S3. UV-vis spectra of Cu(II)-AcAng1-17 at different pH values. [L] = 1x10^{-3} M, metal to ligand molar ratio 2:1.

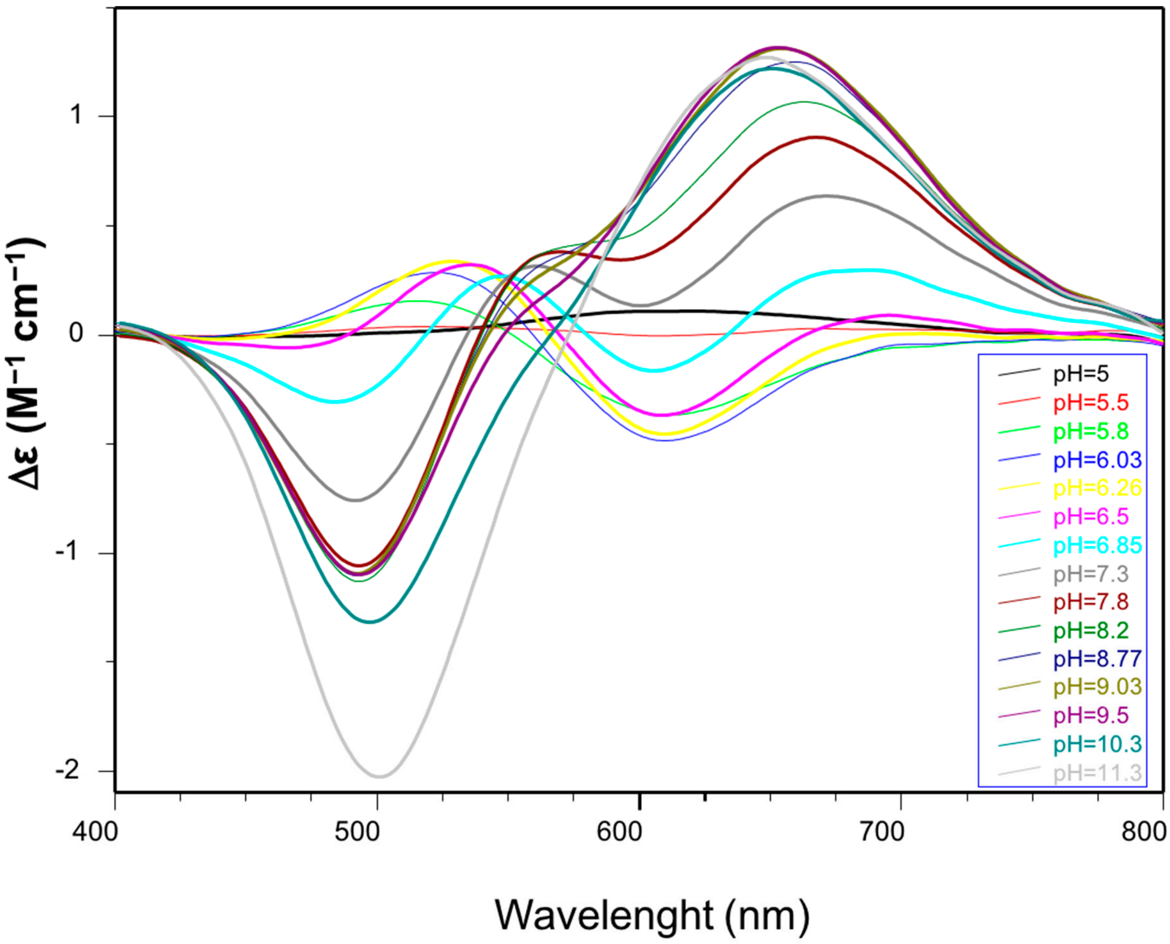

Figure S4. CD spectra of Cu(II)-AcAng1-17 at different pH values. [L] = $1x10^{-3}$ M, metal to ligand molar ratio 2:1.
